# An Overview of Referential Process Linguistic Measures in the Italian Language: Common Elements and Innovative Results

**DOI:** 10.1007/s10936-025-10154-4

**Published:** 2025-05-08

**Authors:** Rachele Mariani, Michela Di Trani, Luigi Solano, Alessia Renzi

**Affiliations:** Department of Dynamic and Clinical Psychology, and Health Studies, Faculty of Medicine and Psychology Sapienza, University of Rome, Via degli Apuli 1, Rome, 00185 Italy

**Keywords:** Mind-Body, Referential Process, Italian Linguistic Measures, Multiple Code Theory, Alexithymia

## Abstract

Language characteristics reflect the ability to connect emotions to words and thoughts, thus transforming non-verbal material into material that can be communicated to others, allowing people to share their emotional experience with others and to regulate their own emotions. In this review, we present the results from the last decade by the three main university research groups (Universities of Rome, Bergamo, and Padua) on Italian computerized linguistic measures of the Referential Process (RP). We discuss 22 studies across several clinical and non-clinical settings and populations, exploring the application of RP measures to heterogeneous materials, such as transcripts of validated clinical instruments, expressive writings, autobiographical memories, dreams, and the therapists’ clinical notes. We also consider the associations between linguistic measures and psychological constructs. The results show the existence of definite linguistic characteristics in the different samples examined. Specifically, a higher use of sensory-somatic words appears to be associated with depressive states, while a greater use of abstract words is associated with defensive dimensions. Additionally, RP measures seem to capture the affective dysregulation features shown by individuals with higher alexithymia scores, indicating reduced symbolizing and affective capabilities. In general, these findings confirm a strong association between language and bodily functioning, highlighting the connection between physical arousal, emotion, thoughts, and health/pathology dimensions.

## Introduction

Bucci (1985, 1997) proposed an integration between cognitive psychology research and psychoanalysis via the Dual Code followed by the Multiple Code Theory (MCT). The MCT has become an important process research model in psychoanalysis. Apart from offering an empirical and innovative approach to study of therapeutic factors, this theory advocates for a unified model of the mind-body relationship. Since its inception, MCT has undergone significant developments, increasing its potential. These advancements are partially due to the application of empirical tools developed within the MCT theoretical framework, which have been employed to study clinical processes and psychopathologies.

In the Italian context, by the late 1990s, the translation of the book, “Psychoanalysis and Cognitive Science” (Bucci, 1997) and the translation of Bucci’s coding manual of Referential Activity (De Coro & Caviglia, 2000) allowed for the establishment of research groups interested in studying the Referential Process (RP). These groups focused on evaluating the effectiveness of psychotherapies and investigating the mind-body relationship in specific clinical populations.

From 2000 to 2010, three research groups were formed in three different Italian universities: Sapienza University of Rome, University of Padua, and University of Bergamo. These research groups began collecting clinical material from sessions and interviews and applied manual coding to evaluate the Referential Process in the Italian language. The collaboration among the three research groups was led by Bucci and Maskit. This allowed the Italian groups to participate almost concurrently with the U.S. group in the development of the most relevant step of this method of investigation: the production of computerized RP language measures.

Referential Activity (RA) is a key study variable linking embodied affective expression with related symbolic processes in the narration of experiences. The most notable step that facilitated the use of empirical tools for assessing RA was the development of computerized methods for applying linguistic measures (Bucci & Maskit, 2007). In 2013, Mariani et al., published the validation of the Italian Computerized Dictionary of Referential Activity, providing an in-depth study of its construction method and a comparison with the English language version. In this paper, we present a review of the main works published in the past ten years regarding the application of RP linguistic measures in the Italian language.

As is well known, Bucci’s MCT (Bucci, 1997, 2021) underscores the significance of the body, non-verbal expressions, and non-symbolic elements in individual functioning processes. It highlights their role in integrating diverse experiences and the transformations activated during psychotherapy. The innovation of MCT lies in proposing codes that allow for the link between non-symbolic affective processes and verbal communication. According to this theory, several elements enable us to move beyond the dualism that has influenced the conception of psychic representation and the definition of illness. From the MCT perspective, the body element closest to the subsymbolic system is a highly organized structure with functional specificities that are interwoven with representational and meaning systems. In this context, the unconscious dimension, as framed by this perspective, takes on new meanings, enabling the exploration of relationships between bodily experiences, symptoms, and their underlying meanings.

According to the MCT, words serve as a primary means to facilitate integration between different processing systems and to communicate with the symbolic and subsymbolic systems of our interlocutors. It is crucial to emphasize that the linguistic style adopted by the speaker, rather than the content of their words, becomes the privileged avenue for understanding the extent to which language is connected to subsymbolic experience. This approach enables us to grasp both the defensive mechanisms employed by individuals to manage internal and external events and an individual’s openness to forming new contents and thoughts (Mariani & Di Trani, 2022).

The examination of the linguistic style employed to articulate emotional experiences, whether spoken or written, offers insight into the activation of the RP. Specifically, the analysis of linguistic characteristics helps to identify the emerging method of connection between words and affective experience. Bucci has categorized these phases of this connecting process as *Arousal*, *Symbolization*, and *Reflection/Reorganization* (Bucci & Maskit, 2022; Solano, 2022).

Bucci and Maskit (2007) have proposed that if the RP fails to develop adequately or is somehow interrupted, such as by traumatic experiences or conflicting dimensions, verbal and non-verbal systems may become disconnected. When this process of disconnection between encoding systems occurs, and the emotional pattern described by Bucci (2021) is activated by an internal or external stimulus, emotional activation may persist in somatopsychic processes in the body, without a connection to verbal systems. This may result in somatic activation or efforts to rationalize or explain emotional events in an abstract way unrelated to the perceived emotional experience, while the symbolization phase, such as a vivid moment of speech or the emergence of a dream, may fail to materialize. This phenomenon is defined by Bucci (2021) as dissociation, “This formulation of dissociation within and between the emotion schemas as underlying emotional disorders is compatible with clinical observations and also with biopsychological data. Van der Kolk (1994) describes the occurrence of fragmentary memories with vivid, intrusive, unmodulated affect, not oriented to space and time, or generalized feelings of anxiety, anger, fear, or uneasiness […] as disconnected images and waves of disjointed sensations and emotions. In multiple coding terms, these are accounted for as dominance of the subsymbolic components of the emotion schema while avoiding acknowledgment of their source” (p. 165). In such cases, language becomes a tool through which specific patterns of dissociation can be analyzed. This includes examining the use of rationalization or abstract language in the narration of personal events (Mariani & Hoffman, 2021; Negri & Ongis, 2021; Renzi et al., 2020a, b).

Bucci has recognized the potential of language as an indicator of internal events, and has consequently developed a suite of computerized linguistic measures, meticulously crafted and validated in Italian. This collection includes computerized linguistic dictionaries (Mariani et al., 2013; Negri et al., 2018) and a self-report questionnaire that empowers therapists to track the progress of the referential process at the conclusion of each session (Negri, Andreoli, Mariani, Negri et al., 2020a, b). These measures are specifically designed to identify and measure diverse linguistic characteristics associated with the referential process.

The computerized linguistic dictionaries encompass various measures, each serving a distinct function in capturing the referential process. To illustrate, the activation of the Arousal phase can be discerned using a dictionary known as “Disfluency” (IDF) (Bonfanti et al., 2008). This dictionary comprises a concise collection of repeated and incomplete words, along with filler pauses that individuals employ in directly communicating their feelings. It gauges the effort exerted in speaking, reflecting functions tied to affective arousal, the activation of emotion schemas, motivation, and evaluation. Similarly, the measurement of “Somatic Sense” (Di Trani et al., 2018) can signify the activation of an Arousal phase, encompassing references to bodily experiences strongly influenced by activations of physiological systems and bodily movement. Moving on to the Symbolization phase, it can be assessed through the application of the Italian Weighted Referential Activity Dictionary (IWRAD) (Mariani et al., 2013). This dictionary evaluates the level of RA and emotional involvement based on linguistic style rather than content, thus exemplifying the non-intentional aspects of emotional expression. Lastly, the measurement of the Reflection/Reorganization phase involves the use of the Italian Weighted Reflection and Reorganization List (IWRRL) (Negri et al., 2018). This dictionary assesses the extent to which the speaker reorganizes, reinterprets, and re-symbolizes elements of the narrative during the elaboration of an emotional experience – a process that could be termed “méta,” referring to the capacity for reflective analysis and retrospective contemplation of the experience.

In conclusion, according to Bucci’s MCT, the characteristics of language reflect the capacity to link emotions to words and thoughts, transforming non-verbal content into communicable material. This process allows individuals to convey their emotional experiences to others and facilitates self-regulation of emotions.

We will now discuss the key research findings from the past ten years since the inception of the computerized linguistic measures of the Referential Process (RP) in 2013. These findings were produced by three research groups based at three Italian universities: Rome, Padua, and Bergamo. These results were developed using the Italian linguistic measures of the Referential Process (RP) and applied in different clinical and non-clinical contexts.

## Method

### Search Strategy and Selection Criteria

The review of computerized linguistic measures of the RP is based on publications from three leading research groups in Italy: Sapienza University of Rome, University of Padua, and University of Bergamo. These groups have developed and applied the RP computerized measures in Italian. A total of 22 publications from 2013 to 2023 were collected for this review, each utilizing RP computerized linguistic measures.

## Results

In this section, we present a summary of the 22 studies selected for this review (see Table 1), organized according to the prevalent themes addressed: (a) the mind/body relationship (*n* = 5 studies); (b) validation of linguistic measures (*n* = 2 studies); (c) narratives related to the COVID-19 pandemic (*n* = 5 studies); and (d) the therapeutic process and outcomes. The latter category, the therapeutic process and outcomes, includes the application of the RA dictionaries to various forms of text, such as transcription of validated clinical instruments, expressive writings, dreams, autobiographical memories, and therapists’ clinical notes (*n* = 10 studies).

**Table 1:** Characteristics of Italian studies on RP computerized measures from the less recent to the most recent

Study	Working group	Prevalent theme	Study Design	Significant findings
Mariani et al. (2013). The Role of Non-Verbal Interaction in a Short-Term Psychotherapy: Preliminary Analysis and Assessment of Paralinguistic Aspects.	Padua University group	d) Therapeutic Process and Outcome	Single case study	RP computerized measures were applied to recorded and transcribed sessions finding effective and significant relationships between speech rate and symbolic processes in the clinician-patient relationship.
Mariani and De Coro (2013). Study of a Short-term Treatment by Referential Activity Linguistic Measures.	Sapienza University of Rome group	d) Therapeutic Process and Outcome	Single case study	RP computerized measures were applied to transcribed sessions finding significant correlations in the patient-therapist exchange to explore the specific interaction during sessions
Rocco et al. (2017). Clinical Mutual Attunement and the Development of Therapeutic Process: A Preliminary Study	Padua University group	d) Therapeutic Process and Outcome	Case study	RP computerized measures were applied to transcription of sessions from two short-term psychodynamic treatments one good and one poor outcome treatment. The good-outcome psychotherapy session is characterized by RP indexes indicating a mechanism of clinical attunement that enforces the therapist–patient relationship and promotes the integration of formal thinking processes that affect emotional and cognitive domains.
Di Trani et al. (2018). Alexithymia according to Bucci’s multiple code theory: A preliminary investigation with healthy and hypertensive individuals.	Sapienza University of Rome group	a) Mind/Body relationship	Case-control	RP computerized measures applied to transcription of alexithymia interviews of students’ and arterial hypertensions patients’ showing a general negative association between somatic sense and alexithymia total and factors scores. In arterial hypertension groups distinct association between peaks of referential activity index and alexithymia emerged
Negri et al. (2019). Linguistic features of the therapeutic alliance in the first session: A psychotherapy process study.	Bergamo University group	d) Therapeutic Process and Outcome	Cross-sectional study	RP computerized measures applied to the transcription of a therapeutic session showing that patients who were rated as more emotionally engaged in relating their experiences and then reflecting on them by mid-session had higher scores in the therapeutic alliance by the final part of that same session.
Mariani et al. (2020). Linguistic analysis of autobiographical narratives in unipolar and bipolar mood disorders in light of multiple code theory	Sapienza University of Rome group	a) Mind/Body relationship	Case-control	RP computerized dictionaries were applied to interviews on autobiographical episode transcripts of patients suffering from depression and bipolar disorders and healthy controls, finding a greater use of affect words, especially negatives, in clinical groups compared to controls. Bipolar patients scored higher in somatic sense compared to depressive and healthy individuals.
Mariani et al. (2020). Giving words to emotions: The use of linguistic analysis to explore the role of alexithymia in an expressive writing intervention.	Sapienza University of Rome group	a) Mind/Body relationship	Cross-sectional study (clinical population)	RP computerized measures were applied to expressive writings of women undergoing assisted reproductive treatments. Women with lower alexithymia were characterized by higher levels of referential activity and symbolization and reflection compared to women with higher alexithymia scores. Women with high levels of alexithymia revealed difficulties in symbolization compared to others.
Negri et al., (2020). Linguistic Markers of the Emotion Elaboration Surrounding the Confinement Period in the Italian Epicenter of COVID-19 Outbreak	Bergamo University group	c) Narrative on COVID-19	Cross-sectional study (general population)	RP computerized measures were applied to expressive writings collected during the pandemic showing higher referential activity, lower abstract reflection, and more positive covariation between referential and reflection/reorganization activities in participants with a friend/relative infected by the virus compared to others. A positive association between themes related to death/religion and referential activity and its covariation with reflection/reorganization activity emerged. A negative association between perceived psychological well-being and reflection/reorganization activity emerged.
Negri et al., (2020). First Validation of the Referential Process Post-session Scale – Therapist version (RPPS-T).	Bergamo University group	d) Therapeutic Process and Outcome	Cross-sectional study	RP computerized measures were applied to the transcription of a therapeutic session and the indexes were compared to those of Referential Process Post-Session Scale – Therapist Version applied to the same session, indicating a substantial convergence between the constructs measured by the two instruments, especially for the symbolizing phase.
Negri and Ongis (2021). Stimulus Features of the Object Relations Technique Affecting the Linguistic Qualities of Individuals’ Narratives.	Bergamo University group	b) Validation of linguistic measures	Cross-sectional study (general population)	RP computerized measures were applied to transcription of responses given to Object Relations Technique cards showing that cards with better form definition and with fewer silhouettes of people elicited responses that were higher in IWRAD and lower in IWRRL, as well as higher in the degree to which the two measures varied together.
Mariani et al. (2021a, b, c). Putting into Words the COVID-19 Lockdown Experience: Psychological Symptoms and the Referential Process.	Sapienza University of Rome group	c) Narrative on COVID-19	Cross-sectional study (general population)	RP computerized measures were applied to writings about the lockdown experience finding a positive correlation between the tendency to rationalize and various scales of distress and a negative correlation between high levels of reorganization and reflection on the experience and symptom scales.
Mariani, Monaco, Christian, et al. (2021). Dreaming in quarantine: Linguistic analysis of referential process of dreams during COVID-19 pandemic lockdown	Sapienza University of Rome group	c) Narrative on COVID-19	Cross-sectional study (general population)	RP computerized measures were applied to narratives on dreams during quarantine. Trough cluster analysis three categories emerged corresponding to the three phases of the referential process
Mariani et al. (2021). Narratives of Dreams and Waking Thoughts: Emotional Processing in Relation to the COVID-19 Pandemic.	Sapienza University of Rome group	c) Narrative on COVID-19	Cross-sectional study (general population)	RP computerized measures were applied to narratives on dreams during quarantine and thoughts on the lived condition during the lockdown finding that Referential Activity was higher in dreams narratives while Reflection and Affect indices were higher in waking narratives.
Mariani and Hoffman (2021). Analytic Process and Linguistic Style: Exploring Analysts’ Treatment Notes in the Light of Linguistic Measures of the Referential Process.	Sapienza University of Rome group	d) Therapeutic Process and Outcome	Single case study	RP computerized measures were applied to the notes of a completed case identifying critical and transformative moments, symbolization processes, and abstraction processes, and managing to capture dysfunctional counter-transferential movements.
Mariani et al. (2022). Emotional dysregulation and linguistic patterns as a defining feature of patients in the acute phase of anorexia nervosa.	Sapienza University of Rome group	a) Mind/Body relationship	Case-control	RP computerized dictionaries were applied to interviews on autobiographical episode transcripts of patients suffering from anorexia nervosa and healthy controls. Sensory-somatic, negative affect dictionaries and RP symbolization difficulty measured by IWRAD predicted the psychopathological group.
Negri et al. (2022a, b). Language and Intelligence: A Relationship Supporting the Embodied Cognition Hypothesis	Bergamo University group	b) Validation of linguistic measures	Cross-sectional study (general population)	RP computerized measures were applied to transcription of autobiographical interviews showing that intelligence scores were related to a greater use of sensory somatic dictionary.
Negri et al. (2022). Relationship between countertransference and emotional communication in the counselling process.	Bergamo University group	d) Therapeutic Process and Outcome	Cross-sectional study	RP computerized measures were applied to clinical sessions showing that countertransference reactions were associated with clients’ lower referential process.
Gennaro et al. (2022). The Role of Interpretation in Fostering the Psychotherapy Process: Evidence from a Single Case Study	Sapienza University of Rome group	d) Therapeutic Process and Outcome	Single case study	RP computerized measures were applied to all the therapy sessions finding that specific interpretative modalities correspond to different patients’ processes of affective elaboration. Specifically, an increase in IWRAD_IWRRL values, is encouraged by clinical interpretative modalities focusing on patients’ inner affective states and on the relationship between affects and behavior, as well as interpretative interventions in which the therapist communicates his own viewpoint. A decrement in IWRAD_IREF scores is promoted by interpretative interventions focusing on events happening inside and outside the session, as well as on the relationship between affects and behavior.
Rocco et al. (2022). The Functional Psychotherapy Approach: A Process-Outcome Multiple Case Study.	Padua University group	d) Therapeutic Process and Outcome	Cross-sectional study	RP computerized measures were applied to psychotherapy session transcripts finding in eight out of ten patients a positive evolution of the therapeutic process showed by WRAD and WRRL scales increased during the sessions and/or as having both over their threshold, as well as a negative value for the REF/WRAD covariation scale.
Mariani et al. (2023). Referential processes in dreams: A brief report from a COVID-19 dreams analysis.	Sapienza University of Rome group	c) Narrative on COVID-19	Cross-sectional study (general population)	RP computerized measures were applied to narratives on dreams and three clusters of dreams coherent with Multiple Code Theory emerged.
Fortunato et al. (2023). Computerized linguistic analysis of counselors’ clinical notes in a university counseling center: Which associations correspond with students’ symptom reduction in a brief psychodynamic intervention?	Sapienza University of Rome group	d) Therapeutic Process and Outcome	Cross-sectional study	RP computerized measures were applied to counselors’ clinical notes to explore differences between the first and the last session finding in the last session a reduction in words, sensory-somatic, negative, and neutral affect words, and an increment in positive affect as well as in Reflection/Reorganization.
Renzi et al. (2023). Women’s Narratives on Infertility as a Traumatic Event: An Exploration of Emotional Processing through the Referential Activity Linguistic Program	Sapienza University of Rome group	a) Mind/Body relationship	Case-control	RP computerized measures were applied to transcription of autobiographical interviews on parenthood journey of women facing fertility issue and women with a child. A greater use of intellectualization and defences in all the narratives associated with the group of women with fertility problems compared to controls emerged.

### The Mind/Body Relationship

Studies included in this section are exclusively from the research group at Sapienza University of Rome, which has published five papers on this topic between 2018 and the present.

Di Trani et al. (2018) explored the correlation between alexithymia and RP measures by applying referential activity computerized dictionaries to transcripts of the Toronto Structured Interview for Alexithymia (Bagby et al., 2006; Italian version Caretti et al., 2011). The findings revealed an inverse association between overall alexithymia scores and the “Somatic Sense” measure, indicating a challenge for individuals with elevated alexithymia levels in utilizing bodily experiences as the primary means of accessing the process of identifying and describing emotions. Moreover, an intriguing finding emerged when the research sample was divided into general population and clinical groups, based on the higher levels of alexithymia observed in hypertensive individuals compared to controls. In the control group, inverse correlations between alexithymia and referential activity remained consistent. However, in the hypertension group, a positive association was observed between peaks of referential activity (Referential Activity Intensity Index) and alexithymia. This suggests that language can reflect a facet of emotional dysregulation, characterized by oscillations between moments of restrained emotional expression and instances of excessive and intense expression. These findings highlight the possibility that different clinical conditions may involve distinct patterns of emotional regulation, which can be effectively discerned through language.

In Mariani et al. (2020), the RP computerized dictionaries were applied to interviews on autobiographical episode transcripts (Relationship Anecdotes Paradigm Interview-RAP, Luborsky, 1998) of patients with mood disorders. The results showed that globally, patients with mood disorders more frequently use “affect” words, especially those denoting negative emotions, compared to a non-clinical group. However, patients with bipolar disorder specifically obtained higher scores in the “Somatic Sense” measure and the Referential Activity Intensity Index compared to the other groups. In this study, the measure of Somatic Sense emerges as a differentiating factor, indicating that patients with bipolar disorder make more pronounced use of somatic experiences, body parts, or symptoms to express their emotions.

An additional study (Mariani et al., 2020) applied the RP computerized dictionaries to the expressive writings of women undergoing assisted reproductive treatments (ART). The results indicated that the writings of women with low levels of alexithymia were characterized by higher levels of referential activity and symbolization and reflection. This suggests a process of meaning-making that progresses from emotion to symbol, and then to reflection - grounded not in abstract terms, but in the context of a specific moment in their lives. Conversely, the writings of women with high levels of alexithymia revealed difficulties in symbolization, which hindered their ability to reflect on emotional experiences. Mariani et al. (2022) highlighted that RP linguistic measures applied to RAP interviews predicted clinical group membership. In particular, the sensory-somatic dictionary, the Negative Affect dictionary, and RP symbolization difficulty, as measured by IWRAD, were significant predictors of membership in the psychopathological group.

A study by Renzi et al. (2023) compared the linguistic features of narratives from women undergoing Assisted Reproductive Technology (ART) treatment with those of women not suffering from fertility problems. The autobiographical interviews examined neutral, positive, and negative episodes in both groups, with a focus on infertility experiences for the ART group and the most difficult moment during pregnancy for the control group. The results highlight a linguistic style characterized by a greater use of intellectualization and defense mechanisms across all narratives associated with the group of women with fertility problems compared to the women in the control group. This suggests that the traumatic and painful experience of infertility and related medical treatment significantly influences the overall manner in which these women narrate their life experiences.

### Validation of Linguistic Measures

The studies included in this section are exclusively from the University of Bergamo research group, which has advanced this area of investigation in recent years through two key papers.

Negri and Ongis (2021) analyzed the stories that participants told elicited by the Object Relations Technique (ORT) card images using RP computerized linguistic measures. The results revealed that cards with better form definition, including color definition and fewer silhouettes of people, elicited responses characterized by higher scores in measures of the connection between language and nonverbal experience (IWRAD) and lower scores in measures of the re-elaboration of emotional experience (IWRRL). Additionally, there was a stronger covariance between these two measures in such responses.

In a related study, Negri et al. (2022a, b) administered an intelligence test and the Relationship Anecdotes Paradigm Interview, analyzed with RP computerized measures to nonclinical subjects. Findings revealed that intelligence scores were associated with the use of words describing somatic and sensory sensations in autobiographical narratives, supporting the hypothesis that sensorimotor schemas play an intrinsic role in language and cognition.

### Narratives on the COVID-19 Pandemic

A specific section is dedicated to studies conducted during the COVID-19 pandemic, considered to be a collective traumatic event that prompted individuals to mobilize their own coping resources. This section encompasses five papers from Sapienza University and the University of Bergamo.

The first study, led by Negri et al., (2020), employed RA dictionaries to analyze expressive writing samples collected during the pandemic lockdown. Results indicated a high level of emotional involvement and the ability to narrate emotional experiences, as demonstrated by above-average referential activity levels in all narratives collected (measured using IWRAD and HPIWRAD indices). Participants were also able to activate a process of reorganization of the experience, exhibiting a capacity for introspection. Moreover, participants who experienced the infection or loss of a friend or relative due to COVID-19 displayed higher referential activity, lower abstract reflection, and a more positive covariation between referential and reflection/reorganization activities, indicative of better emotional elaboration compared to others. Narratives addressing topics related to death, religion and existential concerns showed a high positive correlation with referential activity and its covariation with reflection/reorganizational processes. In addition, it is shown that reflective and rational processes are positively correlated with a lower perception of well-being. This finding indicates a greater need to activate cognitive processes to explain malaise and defend oneself from the most painful experiences.

Mariani et al. (2021a, b, c) aimed to explore the relationship between the linguistic characteristics of writings about the lockdown experience in individuals from the general population. The results showed a positive correlation between the tendency to rationalize in writings and various scales of distress, including depression, anxiety, obsessive symptoms, hostility, and paranoid ideas. Conversely, narratives marked by high levels of reorganization and reflection on the experience showed a negative correlation with distress scales. Language analysis allows for an understanding of how people faced the emotional impact of the pandemic, emphasizing the connection between complex defense mechanisms, such as intellectualization of negative emotions, and psychological symptoms.

A further study conducted during the lockdown period analyzed narratives from the “Dreams Container,” a virtual platform for writing and sharing dreams anonymously during the lockdown (Mariani, Monaco, Christian et al., 2021). Approximately 100 dreams from university students were analyzed using RP computerized linguistic measures. Through cluster analysis, three dream groups emerged, representing the three phases of RP. Specifically, some dreams activated emotion schemas and demonstrated a higher level of emotional arousal (i.e. nightmares). Another group was characterized by high representability and symbolization of the pandemic experience (i.e. claustrophobic imagery, flooding tides, etc.). The third group included dreams focused on cognitive reprocessing, attempting to explain the exhausting and inevitable pandemic experience. In accordance with prior literature, the study suggests that trauma doesn’t alter the elaborative and productive processes of dreams. However, it does impact individuals’ ability to cope with the lingering fear of distressing dreams that carry over into wakefulness (Giovanardi & Spangler, 2021; Saita et al., 2021; Varvin et al., 2021).

Parallel studies (Mariani et al., 2021) compared the texts of the above-mentioned dreams with the individuals’ about their lived experiences during the lockdown. Both types of text were analyzed using RP linguistic measures and a computerized index of Affective Salience (Gennaro et al., 2020). Referential Activity was higher in dreams, highlighting the function of dreams in symbolization, while Reflection (rational and cognitive explanation) and Affect indices were higher in waking narratives. The Affective Salience index was equally high in both types of texts, suggesting the use of two symbolization processes: trauma processing in dreams and the use of emotionally detached language as a defensive mechanism in waking thoughts. This aligns with Bucci’s (2021) concept of emotional schema, where traumatic dimensions activate an excess of distress that cannot be contained by the elaborative processes, leaving the individual unable to represent the experience either in images or in words, often resulting in waking and acting.

More recently, Mariani et al. (2023) applied RP measures to 613 dreams. Three specific clusters consistent with MCT emerged: Cluster A (*N* = 255) defines arousal activation; Cluster B (*N* = 121) defines a phase of symbolization; Cluster C (*N* = 237) defines a phase of reflection/reorganization. Each cluster exhibited specific content and linguistic styles. The arousal cluster, in particular, revealed a pattern of disconnection associated with unprocessed experiences of the pandemic that individuals were trying to confront.

### Therapeutic Process and Outcome

One of the main areas of application of MCT is the study of the clinical process, which aims to understand both the micro-process within individual therapy sessions and the macro-process of the whole treatment. Over the last decade, ten studies by Italian research groups have investigated elements in the clinical exchange that can promote successful treatment outcomes.

A special issue (Rocco, De Bei et al., 2013), explored the application of empirical instruments on the same clinical case. In this short-term treatment, computerized measures of RP in the Italian language were used to examine the relationship between nonverbal and paraverbal aspects and the symbolic and sub-symbolic dimensions of language. Mariani et al. (2013) analyzed linguistic measures and speech rate parameters within recorded and transcribed sessions of a single case, finding effective and significant relationships between speech rate and symbolic processes in the clinician-patient relationship. Similarly, Mariani and De Coro (2013) analyzed macro-processes in a successful case, finding significant correlations in the patient-therapist exchange that highlighted specific dynamics during sessions.

Rocco et al. (2017) compared the micro-processes of two sessions from two short-term psychodynamic treatments – one with a good outcome and the other with a poor outcome. Using two dynamic structural equation models, the study examined clinical attunement as a factor influencing the assessed clinical dimensions. The models fit the data and suggested that the nonverbal attunement played a distinct moderating role. The obtained results are consistent with the hypothesis claiming that a good-outcome psychotherapy session is characterized by a mechanism of clinical attunement that enforces the therapist–patient relationship and promotes the integration of formal thinking processes, thereby influencing emotional and cognitive domains.

Negri et al. (2019) investigated the association between RP computerized linguistic measures and indices of therapeutic alliance scores assessed within the first session of 40 patients. Results showed that patients who were rated as more emotionally engaged in describing their experiences and then reflecting on them by mid-session also scored higher on measures of therapeutic alliance. An implication of this study is that the interpersonal factors facilitating the elaboration of inner experience, including elements of warmth, safety, and analytic trust, are critical for fostering the early therapeutic alliance.

Negri et al., (2020) investigated whether the RP could be measured by a questionnaire completed by psychotherapists at the end of sessions. This clinician report instrument operationalized the scales of the Referential Activity measure into itemized formats as a manual measure. The four original scales were Concreteness, Specificity, Clarity, and Imagination. The Referential Process Post-Session Scale – Therapist Version (RPPS-T) identified four factors that did not completely align with the original manual scales. These factors were named: (1) Concreteness and Imagery; (2) Reflection and Reorganization; (3) Specificity; and (4) Vividness of Session Memories. The correlation analysis between the RPPS-T factors and the RP computerized linguistic measures showed that RPPS-T factor, Concreteness and Imagery, was positive associated with IWRAD. Additionally, the RPPS-T Specificity factor positively correlated with the Referential Activity intensity index, (i.e., the RA picks) and negatively correlated with the Reflection/Reorganization intensity index. Moreover, the RPPS-T Reflection and Reorganization factor showed a significant correlation with the RA intensity measure (MHWRAD), but did not show a correlation with the linguistic measures–WRRL and MHWRRL – which are directly and manifestly related to the reflection and reorganization construct. Lastly, all four RPPS-T scales highly and negatively correlated with the proportion of words relating to bodily and perceptual sensations (SenSD).

More recently, RP linguistic measures have been applied to therapists’ clinical notes, that we could hypothesize represent an internal referential process of the analyst, prompted precisely by the patient’s questioning, inquiry, and stimulation of the analyst’s subsymbolic process (Bucci et al., 2012; Hoffman et al., 2013). In an Italian context, Mariani and Hoffman (2021) analyzed linguistic measures in retrospectively reviewed clinical notes of a completed case, identifying critical transformative moments, symbolization and abstraction processes, and dysfunctional countertransference movements.

Negri et al. (2022) investigated the association between RP and countertransference, finding that countertransference reactions correlated with clients’ speech styles characterized by lower referential activity. Dominant, helpless, overwhelmed, and parental countertransference reactions were especially associated with reduced RP.

Gennaro et al. (2022) demonstrated that specific interpretative modalities correspond with different patients’ processes of affective elaboration. Specifically, an increase in IWRAD_IWRRL values - capturing patients’ ability to engage with an affective experiencing process and make sense of previously warded off content - is facilitated by clinical interpretative modalities that focus on patients’ inner affective states and the relationship between affects and behavior. This increase is also encouraged by interpretative interventions in which the therapist communicates their own viewpoint. Similarly, a decrease in IWRAD_IREF scores, indicating the patients’ process of connecting affects and cognition through non-defensive modalities, is promoted by interpretative interventions that focus on events happening both within and outside of the session, as well as on the relationship between affects and behavior.

Rocco et al. (2022) applied the RP computerized measures to the transcripts of psychotherapeutic sessions in order to explore the evolution of the therapeutic process. A positive progression was defined as an increase in WRAD and WRRL during sessions and/or as having both over their threshold, as well as a negative value for the REF/WRAD covariation scale. According to these criteria, eight of the ten psychotherapies analyzed showed positive trends, with scores over the cutoff for WRAD and WWRL in all the considered sessions, indicating that patients’ strong capacity for symbolization from the outset.

In a recent study, Fortunato et al. (2023) applied the RA computerized linguistic analysis to counselors’ clinical notes to explore differences between the first session (T1) and the last one (T2). Results showed a reduction in sensory-somatic, negative, and neutral affect words at T2, alongside increased use of positive affect words. Symbolization (IWRAD) remained above average (0.5) across both T1 and T2, with a marked increase in Reflection/Reorganization (IWRRL) at T2. This study confirmed the role of linguistic analysis in detecting countertransference responses that may hinder emotional elaboration in counseling sessions.

Studies on clinical notes in English (Bucci et al., 2012; Hoffman et al., 2013) corroborate these Italian findings. They emphasize that the quality of clinical reflection is critical for treatment outcomes. Overly theoretical elaborations or excessively abstract reflections can create distance in the therapeutic relationship, negatively impacting treatment at certain stages.

## Discussion

MCT proposes a model that integrates bodily and mental processes without reducing one to the other, suggesting a mode of functioning in which symbolic and non-symbolic systems of processing experience are interconnected. This connection between systems highlights the inextricable intertwining of bodily and mental processes, while acknowledging their distinct characteristics depending on the observational perspective. The present paper offers an in-depth examination of the Italian application of RP computerized measurements, summarized into four areas of application: (a) mind/body relationship; (b) validation of linguistic measures; (c) narratives on the COVID-19 pandemic; and (d) therapeutic process and outcome.

The historical review highlights the significance of these measures in studying the psychotherapeutic process, as evidenced by the fact that this thematic area was the first to be developed and remains the most consistent. Notably, this topic is the only one that includes contributions from all the Italian research groups. Conversely, the mind/body applications of RP measures, as conceptualized by MCT, are unique to the Roman research group, reflecting their specialized expertise. Similarly, the Bergamo research group has focused on associating these measures with intelligence and visual stimuli, produced the first two Italian papers on this topic. The Padua research group has advanced research by integrating non-verbal measures, such as speech rates, with linguistic measures. This innovative research direction has recently been further developed by Maskit and Bucci, resulting in the creation of a new software tool, T-DAAP, which incorporates measures of speech rhythms and intonation patterns (Maskit, 2025).

### Multiple Code Theory and Alexithymia: Overcoming Body/Mind Dualism

Through the first two topics (a and b) outlined in these studies, MCT emerges as a useful model for reorganizing some concepts strongly linked to the contemporary psychosomatic perspective, such as the construct of alexithymia, defined as the difficulty in connecting with and describing one’s emotions. Early clinical observations and subsequent extensive scientific literature confirm the close relationship between this construct and the development of various somatic and mental pathologies, legitimizing alexithymia as a underling cross-symptom factor present in all pathologies.

A lack of connection between systems leaves physiological processes without the homeostatic regulation that thought provides through reflective and cognitive processing of experiences. Although the similarities with MCT are evident, the initial conceptualization of alexithymia falls into the dualistic perspective, where the body (physiological component) is viewed as separate from the mind (cognitive component). Reinterpreting the concept of alexithymia as an expression of dissociation among systems allows us to overcome the mind/body dualism. This approach contributes to clarifying the distinct nature of the outcomes associated with alexithymia (Taylor & Bagby, 2013).

### The Medical Model Vs. the Bio-Psycho-Social Model: the Meaning of Both Somatic and Psychic Symptoms

Regarding topics a, c, and d, the different populations examined (patients with hypertension; patients with bipolar disorder; and patients with infertility difficulties in a long-term treatment) and the time elapsed between the studies, it is essential to emphasize that today we can reinterpret clinical and research experiences only because the MCT has helped outline a framework of meaning for the results obtained and their points of contact. According to MCT, the mind and body can be considered two expressions of individual multisystemic functioning, nullifying the distinction between somatic and mental pathology. It focuses attention on how symbolic and verbal systems manage and regulate subsymbolic components. The dysregulation of emotional activation, captured in the language of hypertensive and bipolar patients, seems to connect to a symptomatic expression characterized by highs and lows (such as blood pressure or the mood of observed patients). The defining element is not so much the symptom itself but what it represents, namely the attempt to express a common affective dysregulation. Giving meaning to the symptom in these terms is relevant for therapeutic purposes, especially in somatization, where the medical model only aims to eliminate the symptom without attempting to reconstruct its meaning in relation to the person’s functioning within their life context. The somatic symptom is considered, in the Multiple Code Theory, an initial attempt to express distress - expressing what is unthinkable or unspeakable, the result of an initial connection between the subsymbolic and symbolic systems. Rather than eliminating the symptom, therapeutic objectives (including medical outcomes) become the possibility of grasping the evolutionary meaning of its emergence and trying to work with the person to build a way of thinking about the underlying distress.

### Considering Language as an Evaluative Element in the Diagnostic Process of Mental and Physical Disorders

In studying clinical populations (topic a), COVID-19 as a traumatic experience (topic c), and the research process (topic d), we have highlighted how specific linguistic patterns are characteristic of clinical disorders. These data have changed the perspective of clinical evaluation and possible assessment tools. Furthermore, the results underscore a close link between affective reregulation/dysregulation processes and symbolic processes. This type of analysis of linguistic processes can facilitate the clinician’s listening and highlight some elements that can increase clinical understanding. From the narratives studied, for example, it emerges that verbalization based on the naming of negative affects or a language centered on the body and symptoms is associated with symbolic difficulties. Let these, therefore, be elements that help us understand where disconnection processes emerge.

These data are consistent with the theoretical framework previously outlined. Being able to assess elements of psychic functioning based on how people speak not only has significant potential applications in psychotherapy, but also opens the possibility of complementing linguistic measures with categorical diagnostic evaluations. This aims to enrich nosographic frameworks with a dimensional perspective related to the multi-systemic functioning of the individual, as proposed by the Multiple Code Theory. The reported studies, conducted in different contexts and on diverse clinical populations using narrative interviews, highlight the applicability of linguistic measures not only in the psychotherapy research process, where they have mainly been applied, but also in validating both the MCT as a metatheory capable of embracing the entire symptomatic expression. Additionally, these studies demonstrate how linguistic measures can be used to analyze and understand the connections between the individual’s functioning systems in relation to context and life experiences.

## Conclusion

In conclusion, our results showed that Multiple Code Theory and the research tools related to this model can help overcome another type of dualism not yet mentioned: the one between psychoanalysis and research. The possibility of new integrations and dialogues also allows for the development of new theoretical models, which are crucial for the reflective growth of analysts themselves. The three Italian groups are developing future research projects, addressing the relationship between projective tools and emotional activation. The Roman group is continuing the study of narratives in clinical contexts and medical pathologies by exploring the relationship with the experience of pain. The Padua group is developing new studies on the therapeutic process using comparative theoretical models. Future studies will strive to continue overcoming the dualism between psychoanalysis and research.
